# The effect of intradialytic exercise on calcium, phosphorus and parathyroid hormone: a randomized controlled trial

**DOI:** 10.1186/s12882-023-03327-7

**Published:** 2023-09-20

**Authors:** Mohammad Ali Tabibi, Kenneth R. Wilund, Nasrin Salimian, Saghar Nikbakht, Mahsa Soleymany, Zahra Roshanaeian, Farzad Nazemi, Saghar Ahmadi

**Affiliations:** 1 Department of Exercise Physiology, Pardis Specialized Wellness Institute, Isfahan, Iran; 2 Department of Kinesiology and Community Health, Division of Nutritional Sciences, University of Illinois, Urbana-Champaign, USA; 3 Department of Research and Development, Pardis Specialized Wellness Institute, Isfahan, Iran; 4 Department of Kinesiology, Pardis Specialized Wellness Institute, Isfahan, Iran; 5 Department of Sport Nutrition, Pardis Specialized Wellness Institute, Isfahan, Iran; 6 Department of Health and Palliative Care, Pardis Specialized Wellness Institute, Isfahan, Iran

**Keywords:** Mortality, Exercise during dialysis, Bone mineral disorders, Cardiovascular disease, Hematological parameters

## Abstract

**Background:**

Patients with kidney failure experience derangements of circulating markers of mineral metabolism and dysregulation of skeletal and cardiovascular physiology which results in high mortality rate in these patients. This study aimed to evaluate the effect of intradialytic exercise on regulation of these abnormalities in patients receiving chronic hemodialysis (HD).

**Methods:**

In this randomized controlled trial conducted in an HD center in Iran, adult patients receiving chronic HD were randomized to intradialytic exercise (60 min) in the second hour of thrice weekly dialysis for 6 months (intervention) or no intradialytic exercise (control). The primary outcomes were serum calcium, serum phosphorous and parathyroid hormone levels. Secondary outcomes were serum alkaline phosphatase and calcium-phosphorous product.

**Results:**

The study included 44 participants randomized to intervention (*n* = 22) or control (*n* = 22). During the 6-month intervention period, significant between-group changes were observed in all primary and secondary outcomes between the intervention and control groups. Statistical analyses reveal a significant increase in serum calcium (*P* < 0.05) as well as a significant decrease in serum phosphorous, parathyroid hormone, alkaline phosphatase and calcium-phosphorous product (*P* < 0.05).

**Conclusion:**

Intradialytic exercise performed for at least 60 min during thrice weekly dialysis sessions improves bone mineral metabolism in adult patients receiving HD. Further studies should focus on observing and comparing the effect of different types of exercise on bone mineral disorders and all-cause mortality in HD patients.

**Trial registration:**

ClinicalTrials.gov Identifier: NCT04916743, Registered on 08/06/2021. Registered trial name: The Effect of Intradialytic Exercise on Calcium, Phosphorous and Parathyroid Hormone: A Randomized Controlled Trial.

**Supplementary Information:**

The online version contains supplementary material available at 10.1186/s12882-023-03327-7.

## Background

In recent years, there has been a rapid increase in the prevalence of patients with end stage kidney disease (ESKD) that require kidney replacement therapy, including hemodialysis (HD), peritoneal dialysis (PD) or kidney transplant [1]. ESKD patients, especially those on HD and PD, experience high rates of functional impairment, morbidity, hospitalization, and mortality [2, 3]. These outcomes are an urgent priority for patients, caregivers and healthcare professionals [4, 5].

The primary etiological factors responsible for these outcomes include cardiovascular disorders (CVD), muscle atrophy and malnutrition [3]. CVD in ESKD patients is associated with traditional risk factors (diabetes, hypertension, sedentary lifestyle) as well as nontraditional factors (anemia, inflammation, abnormal calcium and phosphorus metabolism and oxidative stress) [6]. High levels of calcium, phosphorus, calcium- phosphorus product and parathyroid hormone (PTH) are associated with all causes of mortality in dialysis patients [7]. Hyperphosphatemia is also a common complication in ESKD. As renal excretion of phosphorus decreases, this results in increased serum levels of phosphorus and hyperphosphatemia. Calcium and PTH are phosphorus-dependent. Hyperphosphatemia alone or in combination with hypercalcemia is associated with increased mortality in hemodialysis patients. Phosphate metabolism is regulated by the interaction of the kidneys, bones, and intestines. This balance is impaired in kidney patients, leading to hyperparathyroidism and vascular calcification [8].

Hyperparathyroidism is one of the aggravating causes of anemia in hemodialysis patients, in addition to resistance to erythropoietin treatment. Defects in calcitriol synthesis, which along with PTH stimulates calcium reabsorption, causes hypocalcemia and hyperphosphatemia in dialysis patients. In this condition, there is an increase in PTH synthesis to regulate calcium and phosphorus homeostasis, leading to secondary hyperparathyroidism [9–11].

Abnormal levels of PTH are associated with disordered muscle protein metabolism, which has been linked to increased mortality in these patients [12, 13]. In chronic kidney disease (CKD) patients, especially in chronic HD patients, the respiratory system is also affected because both uremia and high levels of PTH have deleterious effects on lung function [14]. Therefore, HD patients have respiratory disorders and decreased aerobic capacity due to vitamin D deficiency [15] and high levels of PTH [14]. Indeed, the functional capacity in HD patients has been shown to be about 50% of what it is in healthy populations [16].

The regulation of PTH is critical for bone metabolism in healthy individuals as well as HD patients [17], and may be influenced by physical activity [18]. Indeed, numerous studies in healthy individuals indicate that exercise plays an important role in the regulation of this hormone [19–21]. However, little is known about the role of exercise on parathyroid hormone and calcium and phosphorus metabolism in dialysis patients.

## Methods

### Trial design

This study is an open-label, parallel arm, randomized controlled trial with blinded end-points, which was conducted in a medical center in Iran. Recruitment occurred between July 25, 2021 and 10 August 2021.

### Participants

Individuals were eligible to participate in the study after meeting all of the following inclusion criteria: 1) age ≥ 18 years; 2) receiving regular HD 3 times a week; 3) on HD for at least 1 year, 4) absence of a history of myocardial infarction within the past 3 months; 5) permission from their physician to participate; and, 6) had capacity to provide informed consent to participate in the study. Individuals were excluded if they met any of the following exclusion criteria: 1) cardiac instability (angina, decompensated congestive heart failure, severe arteriovenous stenosis, uncontrolled arrhythmias, etc.); 2) active infection or acute medical illness; 3) hemodynamic instability (systolic blood pressure < 90 mmHg or mean arterial pressure < 60 mmHg); 4) labile glycemic control (extreme swings in blood glucose levels, causing hyperglycemia or hypoglycemia); 5) inability to exercise (e.g. lower extremity amputation with no prosthesis); 6) severe musculoskeletal pain at rest or with minimal activity; 7) inability to sit, stand or walk unassisted (walking device such as cane or walker allowed); or, 8) shortness of breath at rest or with activities of daily living (NYHA Class IV).

### Trial procedures

Before starting the study, some educational and motivational posters were installed in the dialysis center so that all patients became familiar with the benefits of exercise and especially intradialytic exercise. Then, the principal investigator described the side-effects of inactivity and sedentary lifestyle to all interested patients. Specifically, the PI encouraged all patients to be active and provided information regarding potential benefits of intradialytic exercise. After providing written informed consent, eligible patients received a baseline assessment. Data were collected on demographic characteristics (age, sex, and time on hemodialysis), primary cause of kidney failure, and comorbidities (atherosclerotic heart disease, congestive heart failure, cerebrovascular accident/transient ischemic attack, peripheral vascular disease, dysrhythmia, and other cardiac diseases, chronic obstructive pulmonary disease, gastrointestinal bleeding, liver disease, cancer, and diabetes). Comorbidities were quantified using Charlson comorbidity index (CCI) established for dialysis patients, which included the underlying cause of kidney failure, as well as 11 comorbidities [22].

Participants were then randomized in a 1:1 ratio to either the intervention group or control group. The randomization sequence was generated by a study biostatistician who was not otherwise involved in the study using a computer-generated random schedule (using Stata 16, Stata Crop, College Station, Tx). Allocation concealment was safeguarded through the use of sequentially numbered, sealed, opaque envelopes by a specified staff member who was not involved in the study.

### Intervention

Subjects in the intervention group performed concurrent intradialytic exercise during the 2nd hour of dialysis (60-min exercise sessions three times a week) for 6 months. The intervention was a combination of aerobic and resistance exercises. Workout time at the beginning was 30 min and gradually increased to 60 min. Each workout session included 5 min of warm-up, aerobic exercises, resistance exercises and finally 10 min of stretching exercises to cool down. The fistula arm was kept stationary thoroughly the exercise session, with necessary precautions taken into consideration. Also, the exercise protocol was not performed on the arm with AV fistula.

Exercises were individualized in a way that matched the level of physical fitness of participants (See Additional File 1). Aerobic exercises consisted of continuously performed specified movements, such as moving legs back and forth, shoulder abduction and adduction (hand without fistula), flexing and extending the knee, internally and externally rotating the leg, and abducting and adducting the leg, in time with a played beat.

The rhythm of continuous movements was adjusted by the beats per minute of the music. This meant that participants had to coordinate the movements of their arms and legs with the beats per minute of the song being played to them. In this way, the speed and intensity of aerobic exercise was controlled by the rhythm. Resistance training was performed in a semi-recumbent position and included exercises for the upper and lower limbs as well as core strength exercises using body weight, weight cuffs, dumbbells, and elastic bands of varying intensity. Chest press, shoulder press, triceps extension, straight arm shoulder flexion, shoulder horizontal abduction, seated row, supine grip, prone grip, neutral grip, bicep curl, leg abduction, plantarflexion, dorsiflexion, straight-leg/bent knee raises, knee extension, and knee flexion were all part of the resistance training program.

All of the exercises were prescribed by an exercise physiologist who also monitored the exercise sessions and helped patients with any questions they had. At the end of each session, the exercise physiologist reviewed the adherence checklists. If a person did not attend an exercise session, a counseling session was held in the presence of a nephrologist and the exercise physiologist. The reason for the individual's non-participation was investigated and the positive effects of exercise were explained through motivational statements. When possible, patients were reminded of previous benefits of exercise they had experienced as an incentive for them to increase adherence with the training.

Participants in the control group did not undertake any specific physical activity during dialysis.

All other pharmacological, dialysis, dietary and management protocols were identical for participants in both groups and remained unchanged during the study. Despite differences in some hematological parameters at baseline, different medications were not used for intervention group. Major medications related to abnormalities in Ca, P and PTH included phosphate binders like lanthanum carbonate calcium and sevelamer hydrochloride, calcitriol, calcimimetic, and Ca supplement. All participants received normal bicarbonate hemodialysis, which was carried out three times a week for an average of 4 h. Volumetric ultrafiltration control was available on all machines. The standard dialysate flow rate was 500 mL/min and blood flow rates were prescribed according to the participant’s needs. Automated methods were applied to perform dialyzer reuse uniformly.

### Blood sampling

Baseline blood samples were collected one day before the start of the exercise session. Exercise began at the mid-week dialysis session. After the end of the 36th session (end of the third month) and after the end of the 72nd session (end of the sixth month), subsequent blood samples were collected the day before the midweek dialysis session. The control group was assessed at the same time points. On a nondialysis day, blood samples were taken from the scalp vein after at least an 8-h fast. Approximately 30 ml of blood were collected and centrifuged for 15 min at 20 °C and 2500 g. Plasma was next pipetted into cryotubes and stored at -80 °C in a freezer that was electronically monitored. All samples were measured in duplicate, in line with the manufacturers' suggested protocol, and within the manufacturer's specified range of acceptable variation and sensitivity.

### Outcomes

The primary outcome measures included changes in serum calcium (mg/dL), serum phosphorous (mEq/L) and parathyroid hormone (pg/mL) over time. Rate of changes of alkaline phosphatase (ALP) (U/L) and calcium-phosphorous product (mg^2^ /dL^2^) were among the secondary outcomes. All outcomes were evaluated at baseline, 3 months and 6 months.

Safety outcomes included all serious adverse events and adverse events.

### Adherence

Intervention adherence was defined as the number of sessions performed divided by the number of sessions offered, multiplied by 100.

### Blinding

Due to the nature of the intervention, it was not feasible to blind participants or study staff.

However, outcome assessors and data analysts were blinded to participants’ treatment allocations.

### Sample size

The sample size was calculated in accordance with a previous study [23], by NCSS PASS 16.0 software. The model was established according to the Repeated Measure ANOVA (bilateral side) for the PTH as main variable, with α = 0.05 and power 1 – β = 0.8.

Assuming an effect size of 0.45 for reduction of PTH and a drop-out rate of 20%, 44 participants (22 per group) were required to provide 80% power.

### Statistical analysis

Data are presented as frequency (percentage) or mean ± standard deviation, depending on data type and distribution. A detailed statistical analysis plan was prepared and completed prior to database lock. Primary and secondary outcomes were evaluated using Repeated Measure ANOVA and the Friedman test. Statistical analyses were performed using IBM SPSS software 25. *P* values less than 0.05 were considered statistically significant.

## Results

**Fig. 1:**
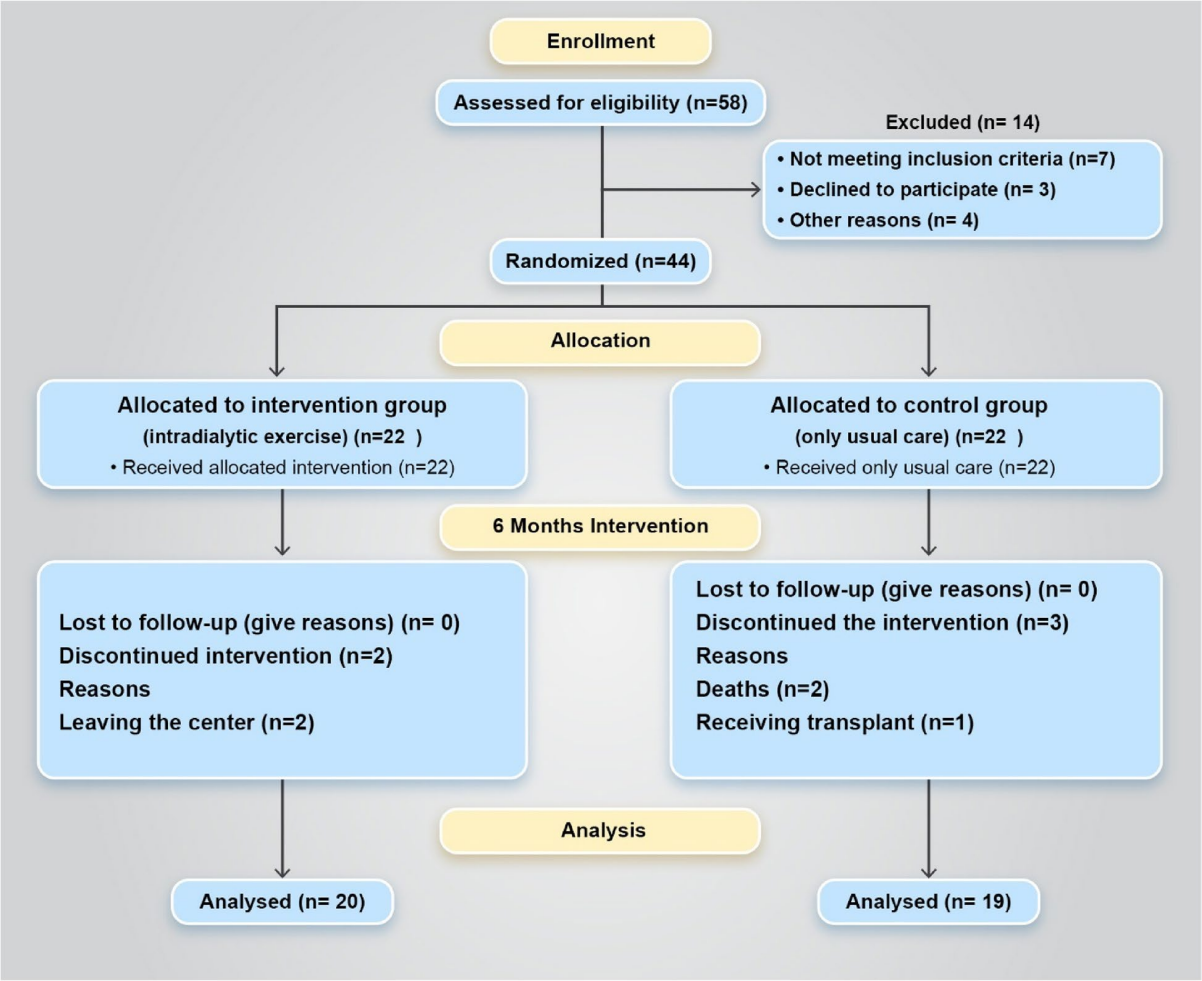
Participant flow during the study

Overall, 58 patients were assessed for eligibility, of whom 44 were consented and randomized. The corresponding flowchart is presented in Fig. 1.

During the 6-month intervention period, 2 participants in the intervention group and 3 participants in the control group dropped out of the study.

### Baseline characteristics

Baseline characteristics were balanced between the assigned treatment groups (Table 1). The adherence rate was 78.5% ± 5%.

**Table 1:** Baseline characteristics of patients

	Intervention (*n* = 22)	Usual Care (*n* = 22)	*P*-Value*
Sex Male	13 (59%)	15 (68%)	0.5
Age (year)	61 ± 14	64 ± 11	0.4
Hemodialysis history (months)	28 ± 11	25 ± 10	0.3
Primary kidney disease			0.8
Diabetes	5 (23%)	3 (14%)	
Hypertension	8 (36%)	9 (41%)	
Glomerulonephritis	6 (27%)	8 (36%)	
Other	3 (14%)	3 (9%)	
CCI	6.5 ± 2.3	5.7 ± 2	0.7

Values are as mean ± (standard deviation) or n (%)

*CCI* Charlson Comorbidity Index

^*^: *P*-value for between group comparisons

### Outcome analysis

Significant between-group changes during the 6-month intervention period were observed in all primary and secondary outcomes (Table 2) between the intervention and control groups. Specifically, serum calcium tended to increase in the intervention group, but remained relatively stable in the control group. In contrast, serum parathyroid hormone, phosphorus, calcium-phosphorous product and alkaline phosphatase levels significantly decreased in the intervention group but remained relatively stable in the control group (Table 2).

**Table 2:** Change of primary and secondary outcomes and the results of longitudinal analysis

	Intervention				Usual care					
	Baseline (*n* = 22)	3 months (*n* = 22)	6 months (*n* = 20)	P**†**	Baseline (*n* = 22)	3 months (*n* = 21)	6 months (*n* = 19)	P**†**	Group × Time	Observed Power
Ca (mg/dl)	8.1 ± 0.6	8.7 ± 0.6	9.2 ± 0.6	< 0.001**	8.4 ± 0.5	8.6 ± 0.6	8.3 ± 0.7	0.4	< 0.001**	0.98
P (meq/lit)	5.8 ± 1.4	5.1 ± 1.1	4.1 ± 0.5	< 0.01**	5.4 ± 1.3	5.9 ± 1.2	5.7 ± 0.9	0.1	< 0.001**	0.97
PTH (pg/ml)	459 ± 192	342 ± 127.4	248.6 ± 78.4	< 0.001**	382.5 ± 166.8	363.5 ± 153.5	375.3 ± 150	0.7	< 0.001**	0.97
Ca × P (mg^2^ /dL^2^)	47.3 ± 11.1	44 ± 8	38 ± 5	0.01*	44.2 ± 10.6	50.4 ± 10	46.2 ± 7.3	0.1	0.003**	0.89
ALP (U/L)	194.6 ± 28.3	161.5 ± 27	135.9 ± 19.5	< 0.001**	200.5 ± 30.4	209 ± 28.3	208.4 ± 31	0.1	< 0.001**	0.99

*Ca* serum calcium, *P* serum phosphorous, *PTH* parathyroid hormone, *ALP* alkaline phosphatase

Values are as mean ± standard deviation

Group × Time is an interaction from a two-way repeated-measures analysis of the effect of time from baseline to 6 months

^†^: ***P***-value for within group changes

^*^p < 0.05 significant

^**^p < 0.01 highly significant

### Safety

No treatment-related serious adverse effects were observed during the period of the study. During the intervention period, one patient had muscle cramps after the first exercise session, but this was not serious or harmful.

## Discussion

The purpose of this study was to examine the effects of intradialytic exercise on calcium, phosphorous and parathyroid hormone. Our primary findings showed that the 6-month intradialytic concurrent exercise program was effective in improving PTH levels, reducing serum phosphorous and improving serum calcium and calcium-phosphorous product as well. However, contradictory results have been reported by some other studies.

Previous work showed that exercise decreased plasma PTH levels in CKD patients [24]. Research on effect of exercise on PTH is limited, but it is affected by physical activity. Its levels can vary after physical exercise depending on the duration and intensity of the activity [25]. Moreover, resistance to PTH action can occur with progression of renal disease [25].

In the present study, phosphorus levels in the intervention group were significantly reduced to lower than the baseline. The effect of exercise on phosphorus level was in line with findings of the several previous studies [26, 27]. For phosphate removal, it appears that intradialytic exercise is helpful. The biphasic regulation of phosphate kinetics during dialysis is explained by a four-pool model in which additional phosphate is produced by erythrocytes and bone as phosphate levels drop [28]. It has been suggested that during intradialytic activity, phosphate is generated by the metabolism of skeletal muscles [29], consequently eliminating the need for extra phosphate from bone and erythrocytes [30]. Phosphate generated from the skeletal muscles after exercise is then used to replenish energy, which results in lower levels of blood phosphate serum [30].

Reviewing the limited available research on the effects of exercise in reducing phosphorus levels showed that although exercise often decreases the level of phosphorus, longer-term exercise and perhaps more intense exercise might be required to see reductions in some studies [26, 31, 32].

This study revealed that serum calcium levels showed significant change in the intervention group, but some other researchers failed to show any influence of exercise on serum calcium [23, 26, 33]. Perhaps studies with longer time frame and differences in type and frequency of exercise are needed.

Renal hyperparathyroidism is a common complication of chronic kidney disease characterized by elevated PTH levels secondary to derangements in the homeostasis of calcium, phosphate, and vitamin D [34].

Abnormalities in calcium, phosphorous, and PTH, hallmarks of the condition known as chronic kidney disease – mineral and bone disorder (CKD-MBD) [35], are associated with adverse outcomes in patients receiving maintenance dialysis [7, 8, 36]. In patients with advanced chronic kidney disease results in derangements of circulating markers of mineral metabolism and in dysregulation of skeletal and cardiovascular physiology [37–39]. Epidemiologic studies of dialysis patients provide substantial evidence that elevated PTH is associated with mortality [8, 36]. However, therapies targeting abnormal CKD-MBD parameters, while improving biochemical end points [40], have failed to convincingly demonstrate reductions in “hard” end points such as all-cause and cardiovascular mortality in clinical trials [41]. The Kidney Disease Improving Global Outcomes guidelines recommend that screening and management of hyperparathyroidism be initiated for all patients with chronic kidney disease [35].

Cardoso et al. in their systemic review found evidence supporting a positive relation between physical activity and bone outcomes in patients with CKD [42]. Studies show that exercise acts as a regulator of homeostasis, alters the levels of circulatory mediators and hormones, and increases the demand for skeletal muscle and other vital organs for energy substrates. Exercise also activates bone and mineral metabolism, especially calcium and phosphate, both of which are essential for muscle contraction, neuromuscular signaling, adenosine triphosphate biosynthesis, and other energy substrates. Due to the fact that PTH is involved in the regulation of calcium and phosphate homeostasis, chronic exercise may help in limiting PTH secretion in exercise activity by changing the level of calcium and phosphate in the bloodstream. On the other hand, the secretion of PTH changes in response to exercise and the resulting myokines, which indicates the systemic effects of physical activity on the secretion of hormones [25].

The present study demonstrated that ALP levels significantly decreased in exercise group, but controls showed no similar change.

Previous observational studies on dialysis patients revealed a relationship between physical activity and serum levels of ALP due to the fact that energy expenditure has a strong relationship to the total BMD [43, 44].

The study by Gomes et al. and the other one by Elshinnawy et al. showed there was significant increase in ALP levels following exercise in patients with CKD [23, 45].

One of the reasons for the contradiction in ALP changes with the previous studies is that in those studies, the ALP level of the patients was within the normal range before the intervention, and the intervention has somehow improved the condition. However, in the current study, the ALP level of the patients was much higher than normal, and exercise has been able to have a significant positive effect on improving the patients' condition by reducing the ALP level.

ALP is an enzyme measurable in most body fluids and usually originates from the liver or bone. In CKD patients without liver disease, ALP is a surrogate of high turnover bone disease and is used to monitor the metabolic bone disease associated with renal insufficiency [46–48]. Furthermore, elevated ALP may be causally involved in the cardiovascular calcification of CKD [49–51], making it a potentially important independent risk factor. Higher ALP has been shown to be associated with mortality and coronary artery calcification in CKD 5D [48] and in patients without CKD [52]. In CKD, ALP is more reliable than many other biochemical bone markers because serum concentrations of ALP are not influenced by the glomerular filtration rate [50]. An association exists between higher serum ALP and worse dual-energy X-ray absorptiometry-assessed BMD in HD patients [53].

Bones are subjected to two sources of loading, including ground reaction force and muscular force [52]. And based on the Mechanostat theory developed by Frost, the local deformation from the mechanical loading stimulates bone cells and results in bone adaptation (i.e., bone tissue accommodates the stress applied on it) [54, 55].

High proinflammatory cytokine levels may contribute to bone loss and fracture in patients with CKD [45]. In addition, inflammatory cytokines are potent activators of receptor activator of nuclear factor kappa B ligand / receptor activator of nuclear factor kappa B (RANKL/RANK)-activated osteoclast genesis [56]. Exercise may inactivate the RANKL/RANK pathway by ameliorating inflammation and preventing bone loss in HD patients [57].

A major strength of the present study was the fact that all exercises were tailored according to each individual's functional status within a pre-specified structure. There was also a high participation rate, despite including HD patients that exhibit considerable diversity with respect to demographic characteristics and associated comorbidities. It is also important to recognize that by the end of the study, levels of calcium, phosphorus and parathyroid hormone in the intervention group reached the level recommended by KDOQI guidelines [35].

This discrepancy between the results of the present study and the findings of the above-mentioned studies may be attributed to differences in participants’ age and sex or duration, intensity, and type of exercise program. In particular, the duration of our intervention was longer than much of what is in the intradialytic exercise literature. Moreover, the intradialytic intervention included both endurance and resistance training components, as opposed to most other studies was performed as concurrent exercise which was different from other studies that include only resistance or aerobic exercise.

Balanced against these strengths, the study also had a number of limitations. Not measuring the effect of exercise on bone mass and cardiovascular indexes are limitations of this study. Indeed, not measuring bone mineral density in this work is a limitation as bone strength depends on bone quality and quantity. Future work should focus on observing and comparing the effect of different types of exercise on bone mineral disorders and all-cause mortality in HD patients.

## Conclusion

Intradialytic exercise performed for at least 60 min during thrice weekly dialysis sessions improves bone mineral metabolism in adult patients receiving HD and perhaps decrease all-cause mortality in these patients. Further studies should focus on observing the effect of different types of exercise on bone mineral disorders and all-cause mortality in HD patients.

### Supplementary Information

**Additional file 1.**

## Data Availability

The data used and/or analyzed during the current study (without any identifying information) are available in Figshare at https://figshare.com and can be accessed with https://doi.org/10.6084/m9.figshare.23551869. The study Protocol also is available in Figshare at https://figshare.com and can be accessed with https://doi.org/10.6084/m9.figshare.22336753.v2.

## Abbreviations

ESKD: End Stage Kidney Disease
CKD: Chronic Kidney Disease
HD: Hemodialysis
PD: Peritoneal Dialysis
CCI: Charlson Comorbidity Index
Ca: Serum Calcium
P: Serum Phosphorous
PTH: Parathyroid Hormone
ALP: Alkaline Phosphatase
CVD: Cardiovascular Disease
CKD-MBD: Chronic kidney disease-mineral and bone disorder
RANKL/RANK: Receptor activator of nuclear factor kappa B ligand / receptor activator of nuclear factor kappa B
GREX: Global Renal Exercise
